# Climate Change and Spatiotemporal Distributions of Vector-Borne Diseases in Nepal – A Systematic Synthesis of Literature

**DOI:** 10.1371/journal.pone.0129869

**Published:** 2015-06-18

**Authors:** Meghnath Dhimal, Bodo Ahrens, Ulrich Kuch

**Affiliations:** 1 Nepal Health Research Council (NHRC), Ministry of Health and Population Complex, Kathmandu, Nepal; 2 Biodiversity and Climate Research Centre (BiK-F), Senckenberg Gesellschaft für Naturforschung, Frankfurt am Main, Germany; 3 Institute for Atmospheric and Environmental Sciences (IAU), Goethe University, Frankfurt am Main, Germany; 4 Institute of Occupational Medicine, Social Medicine and Environmental Medicine, Goethe University, Frankfurt am Main, Germany; University of Liverpool, UNITED KINGDOM

## Abstract

**Background:**

Despite its largely mountainous terrain for which this Himalayan country is a popular tourist destination, Nepal is now endemic for five major vector-borne diseases (VBDs), namely malaria, lymphatic filariasis, Japanese encephalitis, visceral leishmaniasis and dengue fever. There is increasing evidence about the impacts of climate change on VBDs especially in tropical highlands and temperate regions. Our aim is to explore whether the observed spatiotemporal distributions of VBDs in Nepal can be related to climate change.

**Methodology:**

A systematic literature search was performed and summarized information on climate change and the spatiotemporal distribution of VBDs in Nepal from the published literature until December2014 following providing items for systematic review and meta-analysis (PRISMA) guidelines.

**Principal Findings:**

We found 12 studies that analysed the trend of climatic data and are relevant for the study of VBDs, 38 studies that dealt with the spatial and temporal distribution of disease vectors and disease transmission. Among 38 studies, only eight studies assessed the association of VBDs with climatic variables. Our review highlights a pronounced warming in the mountains and an expansion of autochthonous cases of VBDs to non-endemic areas including mountain regions (i.e., at least 2,000 m above sea level). Furthermore, significant relationships between climatic variables and VBDs and their vectors are found in short-term studies.

**Conclusion:**

Taking into account the weak health care systems and difficult geographic terrain of Nepal, increasing trade and movements of people, a lack of vector control interventions, observed relationships between climatic variables and VBDs and their vectors and the establishment of relevant disease vectors already at least 2,000 m above sea level, we conclude that climate change can intensify the risk of VBD epidemics in the mountain regions of Nepal if other non-climatic drivers of VBDs remain constant.

## Introduction

According to the latest report of the Intergovernmental Panel on Climate Change (IPCC), the average warming of the global mean surface temperature was 0.85°C [0.65–1.06°C] over the period of 1880 to 2012 [1]. Importantly, different trends of surface temperature warming at the regional scale and the highest increase has been recorded over the last three decades in mountains and mid-high latitudes of the northern hemisphere[1]. For example, the rate of warming in the Himalayas has been reported to have been much greater (0.06°C/year) than the global average in the last three decades indicating that the Himalayas are more sensitive and vulnerable to global climate change than other areas of the earth [2]. Climate change affects human health mainly by three pathways: (1) direct impacts by increasing the frequency of extreme events such as heat, drought and heavy rainfall, (2) effects mediated through natural systems such as disease vectors, water-borne diseases and air pollution, and (3) effects that are heavily mediated by human systems such as occupational impacts, under-nutrition, and psycho-social problems [3,4]. As the published literature continues to focus on the effects of climate change in developed countries, the effects on the most-vulnerable populations residing in least developed and developing countries are underreported [3]. These poor or developing countries are historically least responsible for greenhouse gas (GHG) emissions but most vulnerable to climate change impacts, and are also currently suffering the heaviest disease burden indicating an “ethical crisis” [5]. Several challenges for conducting climate change and health research in developing mountainous countries have been reported. These include a lack of trained human resources, financial resources, long-term data and information, and suitable methods that are applicable to the local context [6]. Furthermore, the largest health risks will occur in populations that are most affected by climate sensitive diseases such as vector-borne diseases (VBDs) and in those left behind by the economic growth [4].

Most of the VBDs are transmitted by insect vectors and caused by pathogens that circulate in the human or between human and other animal populations [7,8]. There is increasing evidence about the impacts of climate change on VBDs, and some of it can be explained by the fact that the insect vectors of these diseases are ectothermic and hence temperature affects their vectorial capacity and the extrinsic incubation period (EIP) of pathogens [8,9,10]. Additional climate variables like precipitation also play important roles, but their trends under climate change and their biological impact are even less understood than temperature effects alone. Changes in the geographic distributions of insects are often regarded as being among the more readily observable and predictable effects of climate change [11]. However, effects of climate change on human VBDs can be masked by other important socio-economic factors, host immunity, medical care and vector control interventions, and this complexity has given rise to controversies among scientists [12,13]. There arestudies which have documented that climate change is already affecting the distribution of VBDs such as highland malaria [14,15,16,17]and many studies have predicted direct effects of climate change on malaria [14,15,18,19,20,21,22,23], dengue [24,25,26] and leishmaniasis [27]. In spite of these observations and projections, there are reservations about the possibility to model the future transmission of VBDs under climate change scenarios, and several authors have pointed out the need for understanding the epidemiology of VBDs using alternative hypotheses that are not climate driven [28,29,30,31,32,33,34,35,36,37,38,39].

Although no single factor can fully explain the transmission of VBDs, climate change can alter the geographical distribution of disease vectors and VBDs [3], for example, by rendering previously endemic areas unsuitable and previously non-endemic areas suitable for their existence and reproduction [40,41,42]. As higher latitudes and altitudes are more sensitive to climate change and experiencing higher warming rates [2,43,44], a shift of species distributions towards higher elevations has been predicted [45]. Several recent studies have shown an increasing trend of the epidemic potential and length of the transmission season of VBDs in temperate regions and tropical highlands under different climate change scenarios [14,18,19,24]. Moreover, the IPCC concludes that local changes in temperature and rainfall will continue to alter the distribution of disease vectors and the risk of VBDs [4], and that the population mostly affected by climate-sensitive diseases and is deprived of participation in economic growth will face the largest health risk [4,46]. However, heterogeneity in VBD transmission is observed at every spatial scale ranging from less than a kilometre to continents. This heterogeneity is determined, for example, by the ecology and biogeography of the vectors, soil types, urbanization, local adaptation to temperature, and host communities [47,48]. Therefore, evidence generated at the global level may not be applicable at a local scale, and understanding the spatiotemporal distribution of VBDs in an individual country is important.

Nepal is one of the most vulnerable countries with respect to climate change because it is positioned in the southern rim of the so-called “Third Pole” of our planet (the Himalaya-Hindu Kush mountain range and the Tibetan Plateau). It has a complex topography and a low level of development [49]. Despite its largely mountainous terrain, Nepal is afflicted with five VBDs, namely malaria, lymphatic filariasis (LF), Japanese encephalitis (JE), visceral leishmaniasis (VL) (also known as kala-azar) and dengue fever (DF)[50]. Most of the major VBDs included in this study may be considered as neglected tropical diseases (NTDs). Their characteristics in Nepal are provided in Table 1.

**Table 1:** Characteristics of vector-borne diseases in Nepal.

Diseases	Pathogen	Reservoir	Principal vector	Reported confirmed cases in 2012^a^	References
**Parasitic**					
Malaria	*Plasmodium falciparum*, *Plasmodiumvivax*	Humans	*Anopheles fluviatilis*, *Anophelesannularis*, *Anopheles maculatus* complex	2,092	[51,52]
Lymphatic filariasis	*Wuchereria bancrofti*	Humans	*Culexquinquefasciatus*	28,855	[52,53]
Visceral leishmaniasis (Kala-azar)	*Leishmania donovani*	Mammals	*Phlebotomus argentipes*	575	[54,55,56]
**Viral**					
Dengue fever	Dengue virus (Flaviviridae)	Humans	*Aedes aegypti*, *Aedesalbopictus*	183	[57,58,59]
Japanese encephalitis	Japanese encephalitis virus (Flaviviridae)	Birds, pigs	*Culex tritaeniorhynchus*	129	[52,60]

^a^Reported cases to the Epidemiology and Disease Control Division [50].

The Nepal National Adaptation Programme of Action (NAPA) to climate change has identified VBDs as one of the highest priority adaptation projects forthe health sector in Nepal [49]. The higher warming trend in the hill and mountain regions of Nepal and increasing numbers of autochthonous cases of VBDs reported from non-endemic hilly and mountain regions motivated us to conduct this review. By reviewing the published literature we wished to explore whether these observed spatiotemporal distributions of VBDs can be attributed to climate change. Thus, this article aimed to review the available literature on VBDs and climate change to allow for an assessment of the likely impacts of climate change on the changing spatiotemporal distribution of VBDs in Nepal.

## Methods

A systematic literature search was performed and summarized information on climate change and the spatiotemporal distribution of VBDs in Nepal following providing items for systematic review and meta-analysis(PRISMA) guidelines. We searched for peer-reviewed articles published in English language before December2014 in the PubMed and Web of Science databases. Besides, we searched for relevant journal articles in Google Scholar and retrieved government reports from their web sites. We used the following search terms in title, abstract and keywords:

1. Nepal and
2. Climate, climate change, temperature, rainfall, precipitation, relative humidity, weather, *Aedes*, *Anopheles*, *Culex*, *Phlebotomus*, dengue, malaria, kala-azar, Japanese encephalitis, visceral leishmaniasis, lymphatic filariasis, mosquito-borne diseases, VBDs.

We first screened “titles”, “abstracts” and “keywords” for relevant articles and then read full text articles to evaluate them according to our inclusion criteria. Furthermore, the reference lists of each selected research article were then evaluated using the snowball sampling technique if they had been missed in the electronic databases. Inclusion criteria for selecting studies are listed below:

### Inclusion criteria

1. Studies must include climatic variables (Rainfall, temperature and humidity) that analyse the trend of climatic data and are relevant for the study of VBDs
2. Epidemiological studies dealing with the spatial and temporal distribution of disease vectors and disease transmission and/or epidemiological studies assessing the association of VBDs with climatic variables
3. Only studies published before December2014 and with a study area in Nepal

The selected papers were systematically reviewed and thematically analysed. Conference proceedings, viewpoint articles, review articles, project reports and theses were excluded. Given the low number of studies meeting the inclusion criteria and their mostly descriptive nature, a quantitative meta-analysis was not appropriate. Therefore, we alternatively summarized the state of the art on climate change and the spatiotemporal distribution of VBDs in Nepal. The spatio-temporal distributions of VBDs from the published literature was projected onto a map of Nepal using GIS software.

## Results

### Literature search

**Fig 1:**
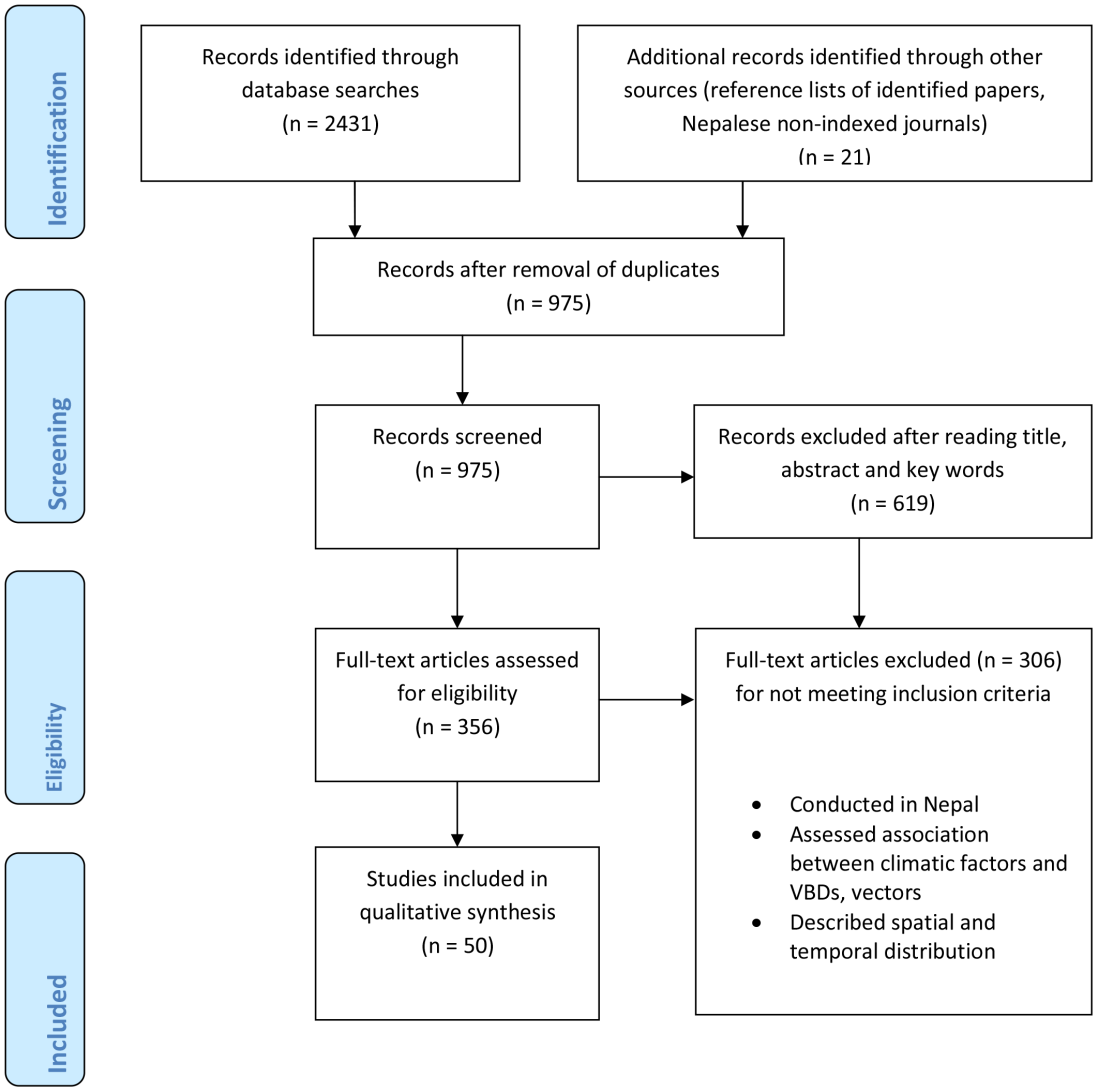
PRISM flow diagram of the literature search.

The preferred PRISM flow diagram of our literature search is given in Fig 1, and a summary of relevant papers retrieved for evaluating their inclusion in Table 2.

**Table 2:** Summary of full text articles retrieved and identified for qualitative synthesis.

Topics	Full-texts retrieved	Included in qualitative synthesis^a^
Climate change	65	12
Malaria	98	8
Lymphatic filariasis	8	7
Visceral leishmaniasis (Kala-azar)	110	8
Dengue fever	27	9
Japanese encephalitis	45	11
Climatic factors and VBDs	8	8

^a^Some studies dealt with more than one diseases and their vector. Hence, the total number of final studies included in the qualiatitive synthesis was 50 (Tables 3 and 4).

We found 12 studies that analysed the trend of climatic data and are relevant for the study of VBDs, 38 studies that dealt with the spatial and temporal distribution of disease vectors and disease transmission. Among 38 studies, only eight studies associate climatic factors with VBDs in Nepal. Among these eight studies, one dealt with malaria and VL, one with dengue and LF, three dealt with malaria and one each with JE, LF and VL.

**Fig 2:**
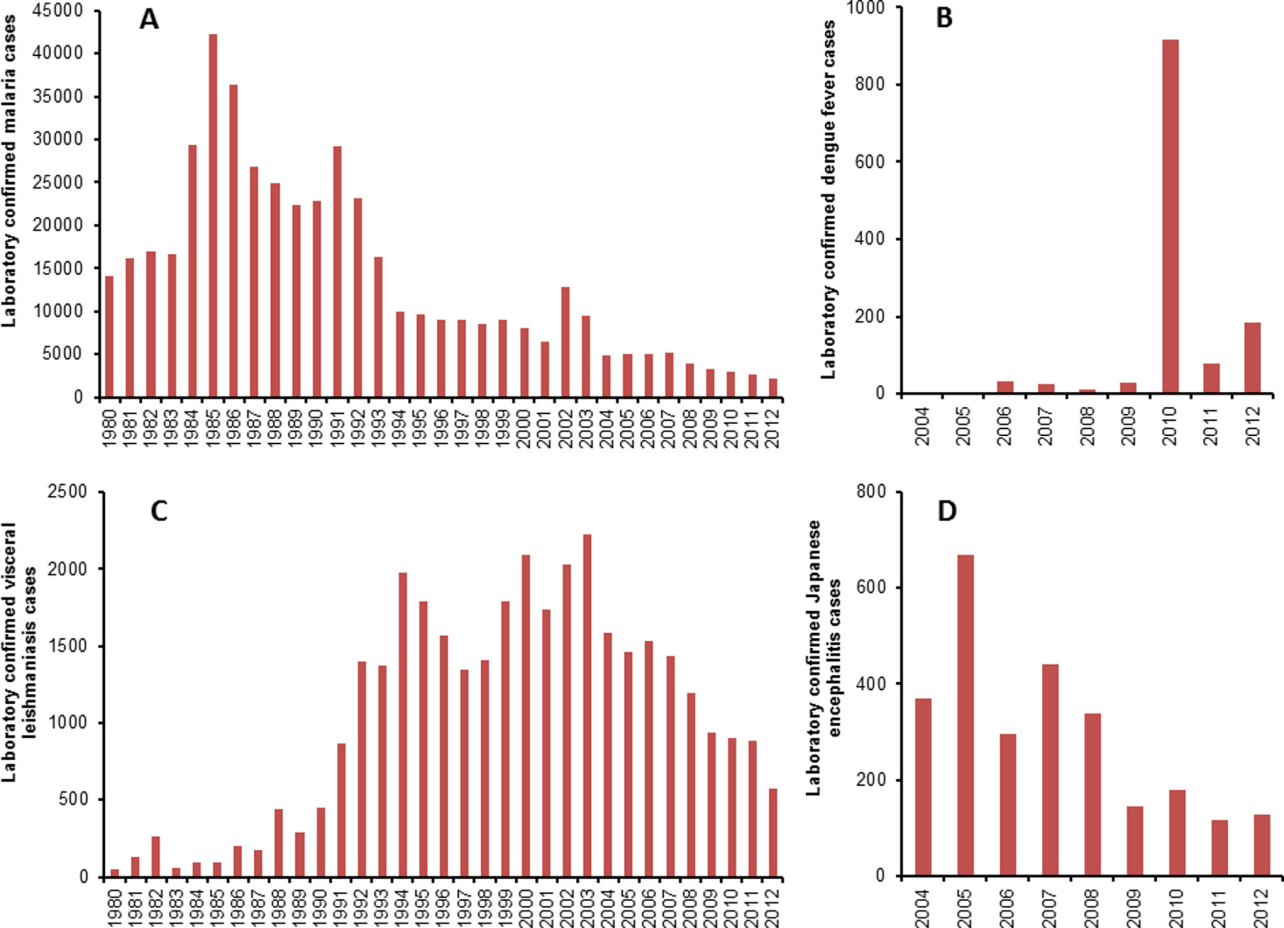
Trend of confirmed cases of vector-borne diseases in Nepal. Panels A, B, C and D show, respectively, the trend of confirmed malaria, dengue fever, visceral leishmaniasis and Japanese encephalitis cases reported to the Epidemiology and Disease Control Division, Department of Health Services, Ministry of Health and Population, Government of Nepal.

A summary of the main findings from the analysis of climatic variables is provided in Table 3. Similarly, a summary of the main findings of studies on the spatiotemporal distribution of VBDs and their association with climatic variables is presented in Table 4. The trend of confirmed cases of major VBDs except LF reported annually to the Epidemiology and Disease Control Division (EDCD) of Nepal’s Ministry of Health and Population is presented in Fig 2. This Fig 2 shows declining trend of VBDs except dengue in Nepal especially in endemic areas where diseases control programmes are intensified by the Government of Nepal with support of external development partners. Despite these declining trends due to human interventions, VBDs are expanded in new areas which were previously considered non-endemic.

**Table 3:** Summary of findings on analyses of climatic variables.

Study	Location (Study period)	Climatic variables	Method	Main findings	Comments
Shrestha *et al*. 1999 [61]	Nepal (1971–1994)	Maximum temperature	Trend analysis based on observed station data	Higher warming rates in high-elevation areas (mountain and Himalayan regions) compared to lowlands (Terai and Siwalik hills). Warming rates were highest in the post-monsoon season followed by winter	The rate of warming in Nepal shows features similar to that of the northern hemisphere but its warming rate is much higher than the global average
Shrestha *et al*. 2000 [62]	Nepal (1948–1994)	Rainfall	Trend and spectral analysis based on observed station data	Monsoon precipitation series shows great interannual variability and decadal variability in the amount of precipitation without any distinct trend	Precipitation records from Nepal resemble those of northern India suggesting that all-India precipitation data cannot provide a valid representation of the entire Indian sub-continent.
Kansakar *et al*. 2004 [63]	Nepal (1965–1995)	Precipitation	Cluster analysis based on observed station data	Reports spatial and temporal variation in precipitation pattern and significant roles of mountainous relief in yielding localized precipitation patterns, and precipitation timing is more stable than its amount.	Precipitation in Nepal is broadly contributed by monsoon and western disturbances
Baidya *et al*. 2008 [64]	Nepal (1961–2006)	Temperature and rainfall	Trend analysis based on observed station data	Both temperature and precipitation extremes show increasing trends. Decreasing trends of cool days and increasing trends of warm days are very prominent at higher elevation. Similarly, precipitation extremes show increasing trends in total and heavy precipitation events at most stations	The temperature data of only 8 stations from 1971–2006 and precipitation data of 26 stations between 1961–2006 were included in analyses.
Dobler *et al*. 2011[65]	South Asia (1961–2100)	Precipiation	Regional Climate Models projections	Over 70% decrease in monsoon rainfall in parts of northern India at the end of 21 century is predicted.	Because of higher evapotranspiration rates with higher temperature, a decreasing trend in water availability is possible.
Shrestha *et al*. 2011[66]	Nepal (1977–2000)	Maximum temperature	Trend analysis based on observed station data	The extended analysis also shows an increasing trend of maximum temperature without decrease; warming in winter is more pronounced compared to other seasons	A similar analysis of precipitation data does not reveal any significant trend (precipitation depends on monsoon circulation in Nepal)
Kattel *et al*. 2012 [67]	Nepal (1985–2004)	Temperature	Regression analysis based on observed station data	A bi-modal pattern in the annual cycle of temperature lapse rate is observed (two maxima in the pre-and post-monsoon seasons and two minima in the winter and summer season) which are in contrast to results from other mountain regions.	Air temperature records from 56 meteorological stations in Nepal ranging from 72 to 3920 m asl were used for analysis. The temperature lapse rates in the southern slopes of the central Himalayas are in contrast to those from other mountains suggesting that different controlling factors are associated with them.
Shrestha *et al*. 2012 [68]	Central Himalayas (1998–2008)	Rainfall	Spatiotemporal variation analysis based on satellite data	A strong positive relationship between elevation and rainfall during the pre-monsoon season and two significant rainfall peaks during the summer monsoon season were found over the southern slope of the Himalayas; the primary peak along the Sub-Himalayas (~500–700 m asl) and the second peak along the Lesser Himalayas (~2,000–2,200 m asl)	Only data from the pre-monsoon and summer monsoon seasons were included in analyses; the highest altitude considered was 5,000 m asl.
Kattel *et al*. 2013 [69]	Nepal (1980–2009)	Temperature	Trend analysis based on observed station data	A statistically significant average warming trend with a prominent rise of maximum temperatures is found while minimum temperature trends show greater variability among stations	Only 13 mountain stations ranging from 1304–2566 m asl were included for analysis
Kulkarni *et al*. 2013 [70]	Hindu-Kush Himalayan (HKH) Region (1961–2098)	Temperature and rainfall	Regional Climate Models projections	The model projections indicate significant warming throughout the HKH region towards the end of the 21st century. For 2011–2040, temperature is projected to increase by 0.5–1°C; for 2040–2070, by 1–3°C and for 2071–2098, by 4–5°C. In contrast, precipitation projections indicate a declining trend of monsoon precipitation especially in the Central Himalayas between 2011–2070 compared to the baseline period 1961–1990	The high resolution regional simulations were generated using the regional climate model PRECIS and were validated using observedAPHRODITE precipitation data and NCEP/NCAR temperature data.
Qi *et al*. 2013 [71]	Mt. Qomalangma (Everest) Region in Nepal (1971–2009)	Temperature and precipitation	Trend analysis based on observed station data	A significant increase in annual mean temperature (0.025°C/year) highly influenced by maximum temperature was found. Similarly, annual precipitation shows an increasing trend (4.77 mm/year) with concentration of precipitation mainly in the monsoon period.	In contrast, the northern slope temperature rise is highly influenced by minimum temperature and precipitation is many times lower compared to the southern slope indicating a barrier effect of the Himalayas.
Aryal *et al*. 2014 [72].	Mustang, Nepal (1987–2009)	Temperature and precipitation	Regression analysis based on observed station data	Maximum and minimum temperature have increased over the years with a significant average increase of the mean annual temperature (0.13°C/year). Similarly, precipitation has increased significantly (0.541 mm/year).	The significant snow melt perceived by local people might be due to combined effects of higher temperature and increased rainfall.

**Table 4:** Characteristics of studies on the spatiotemporal distribution of vector-borne diseases and their association with climatic variables.

Study	Location (Study period)	Diseases /vector	Method	Main findings	Comments
Pandey *et al*. 2004 [73]	Nepal (2004)	Dengue	Case report	The first reported case of dengue virus (DENV) infection in Nepal	The case was a Japanese volunteer in Nepal
Malla *et al*. 2008 [58]	Nepal (2006)	Dengue fever and vector	Descriptive	First reported outbreak of dengue fever with confirmation of local transmission by *Aedes aegypti* in nine urban areas of lowland Nepal	All four serotypes of DENV reported to circulate
Shah *et al*. 2012 [74]	Western Nepal (August 2008-November 2009)	Dengue fever	Descriptive	Report of geographical expansion of dengue virus to new areas (western Nepal)	Only sero-prevalence survey
Dumre *et al*. 2013 [75]	Nepal (2005–2010)	Dengue fever	Descriptive	Rapid expansion of dengue fever within a short period of time with confirmation of dengue fever from 18 districts including four hill districts.	There is no dengue fever surveillance network and cases (from referral laboratory) are grossly underreported
Pandey et al. 2013 [59]	Nepal (2010)	Dengue fever	Descriptive	Dengue virus 1 was possibly responsible for the 2010 epidemic and imported from India to the Terai lowlands of Nepal from where it spread to the highlands. The primary vector *Aedes aegypti* was identified in all epidemic areas (12 districts) including four hill districts.	DENV isolation from mosquitoes was not performed
Henderson *et al*. 1983 [76]	Eastern Nepal (1983)	Japanese encephalitis	Descriptive	Report of JEV introduction in southern Nepal in the late 1970s after a major epidemic in northern states of India	High susceptibility of adults is taken as an indicator of a recent introduction of JEV
Zimmerman *et al*. 1997 (2005) [77]	Kathmandu valley (1995)	Japanese encephalitis	Descriptive	Report on first epidemic of JE among local residents of Kathmandu valley in 1995	Records of only one hospital were reviewed excluding children
Bista and Shrestha, 2005 [78]	Nepal (1978–2003)	Japanese encephalitis	Descriptive	JE was endemic in 24 districts of lowlands (Terai and inner Terai) with the majority of cases occurring in the monsoon season	Possible expansion of JE virus in temperate regions is discussed
Partridge *et al*. 2007 [79]	Kathmandu valley (2006)	Japanese encephalitis	Descriptive	Local transmission of JE in Kathmandu confirmed with majority of cases reported during and after the monsoon season.	Laboratory confirmed JE cases were followed up to confirm area of residence and travel history to JE endemic areas
Bhattachan *et al*. 2009 [80]	Nepal (2007)	Japanese encephalitis	Descriptive	JE confirmed as endemic in 21 additional hill and 3 three mountain districts previously considered non-endemic	No detailed information on new JE endemic districts is provided
Impoinvil *et al*. 2011 [81]	Nepal (2004–2008)	Japanese encephalitis	Local Indicators of Spatial Association (LISA) analysis and Spatial lag regression model	The distribution of JE after 2005 has shifted to Kathmandu valley and mountain districts showing a significant negative relationship between JE incidence and April precipitation, and a significant positive association between JE incidence and the percentage of irrigated land, climatic, agriculture and land cover parameter. Before 2006, JE cases were clustered in the lowlands	Only data of 2005 was used to fit a spatial lag regression model with climate, agriculture and land cover as explanatory variables
Thakur *et al*. 2012 [82]	Four mountain districts of Nepal (July-August 2010)	Japanese encephalitis	Generalized linear mixed modelling	Report of JE virus infections among pig populations in high-altitude mountain districts with decreasing risk of seropositivity with increasing elevation	Impact of climate change on the circulation of JE virus in mountain districts is discussed
Robertson *et al*. 2013 [83]	Nepal (2007–2011)	Japanese encephalitis	Geographically weighted regression	Cases were positively associated with a high degree of landscape mixing and small-scale agriculture	A recent trend towards establishment of Japanese encephalitis in the Kathmandu valley and mountain districts was confirmed in this analysis
Jung 1973 [53]	Kathmandu (1972)	Lymphatic filariasis and vector	Descriptive	*Culex quinquefasicatus*, the principal vector of *Wuchereria bancrofti* in Nepal, was recorded within the endemic zone of LF.	Study area was limited to Central Nepal
Scherchand *et al*. 2003 [84]	Nepal (2001)	Lymphatic filariasis	Descriptive	Epidemiological mapping of lymphatic filariasis showed that 33 out of 37 districts were endemic including densely populated districts of Kathmandu valley (~1,400 m asl)	The highest altitude sampled was 1,400 m asl
Adhikari *et al*. 2008 [85]	Four districts of Nepal (Feb-July 2007)	Lymphatic filariasis	Descriptive	The highest microfilaria infection rate and aysmptomatic cases were recorded in mountain districts compared to lowland and hill districts.	Entomological data were not collected
Pradhan *et al*. 1970 [51]	Mugu district (Gum Valley) (1969)	Malaria and vector	Descriptive	Local malaria transmission along with malaria vectors were recorded above 1,800 m in Nepal. The malaria vector *Anopheles maculatus* was recorded up to 3,100 m asl.	Malaria transmission was seasonal
Sakya 1981 [86]	Nepal (1978–1980)	Malaria	Descriptive	Malaria cases were reported from 38 districts out of a total of 75 districts, including the Terai lowlands and hill districts mostly below 1,200 m asl.	Cases were detected under active malaria surveillance and follow-up of all confirmed cases was performed
Dahal 2008 [87].	Nepal (1978–2006)	Malaria and Visceral leishmaniasis	Descriptive	Malaria incidence has declined remarkably over the years but the number of districts where malaria is prevalent increased to 67. A positive relationship between rainfall and malaria cases with a certain time lag (1–2 months) is reported. Similarly, positive relationship between annual mean temperature and rainfall with Visceral leishmaniasis cases is reported	Only a few years monthly data were used to show an association of rainfall and malaria outbreaks. Only few years monthly data of Visceral leishmaniasis was used.
Bhandari *et al*. 2013 [88].	Jhapa district (1998–2009)	Malaria	ARIMA Time series analysis	Significant positive correlations between the climatic variables temperature (minimum and maximum), rainfall and malaria cases were found. However, in time series analysis, climatic variables were not significant predictors of malaria incidence	Non-climatic variables were not included in time series analysis and climatic variables were not significant predictors
Dhimal *et al*. 2014 [89]	Nepal (2004–2012)	Malaria	Generalized linear models	Despite normal seasonality of rainfall and temperature during the study period, the incidence of annual confirmed malaria cases declined significantly in historical lowland high and moderate-risk districts. This coincided with the free distribution of long-lasting insecticidal nets (LLINs) suggesting that effective vector control interventions can outweigh the role of climate. However, the risk of malaria epidemics in highlands is predicted to increase due to climate change.	Malaria, non-malaria (Total outpatients visits per year, childhood diarrheal diseases and acute respiratory infection) and climatic data were analysed.
Dhimal *et al*. 2014 [23]	Kailali and Jhapa districts of Nepal (2004–2012)	Malaria	Generalized additive mixed models	Strong relationship between monthly temperature and malaria incidence is reported. A 1°C increase in minimum and mean temperatures increased malaria incidence by 27% and 25%, respectively. Malaria hotspots persisted mostly in the same villages of Kailali district, whereas in Morang district malaria hotspots shifted to new villages after the introduction of LLINs.	A combined model with both climatic and non-climatic predictors was not developed and net effect of vector-control interventions and climatic factors is not known.
Kakchapati and Ardkaew, 2011[90]	Nepal (1998–2009)	Malaria	Negative binomial regression modelling	A decreasing trend in the incidence of malaria (1998–2004), followed by a more moderate upward trend until 2008 is found. Zero malaria incidences occurred in six districts for over twelve years and higher incidences were reported among districts bordering India except Kavre district.	Only yearly malaria incidence was used without categorizing indigenous and imported cases, or malaria infections by parasite species
Peters and Dewar 1956 [91]	Central Nepal (1954–1955)	Vector	Descriptive	Secondary vector of dengue (*Aedesalbopictus* and principal vector of lymphatic filariasis (*Culex quinquefasciatus*) recorded to range from lowland to hill regions	Highest altitude district studied was Kathmandu
Joshi *et al*. 1965 [60]	Nepal (1956–1965)	Vector	Descriptive	Principal vector of JE, *Culex tritaeniorhynchus*, first recorded in 1963 with distribution from lowlands to Kathmandu (around 1,350 m asl)	The highest altitude surveyed was 1400 m asl; survey confined to eastern and central Nepal only
Darsie and Pradhan 1990 [52]	Nepal (1950–1989)	Vector	Descriptive	The principal vectors of lymphatic filariasis (*Culex quinquefasciatus*), of Japanese encephalitis (*Culex tritaeniorhynchus*)and the secondary vector of dengue virus(*Aedes albopictus*) were documented up to Kathmandu valley (1,350 m asl) in Nepal	The principal vector of dengue virus (*Aedes aegypti*)was not recorded in any survey
Darsie *et al*. 1994 [92]	Mustang district (1993)	*Vector*	Descriptive	Breeding of *Anopheles* mosquitoes was recorded up to 3,738 m.	These *Anopheles* mosquitoes were not malaria vector
Gautam *et al*. 2009 [57]	Kathmandu (April-June 2009)	Vector	Entomological survey	First report of the dengue virus vector *Aedesaegypti* in Kathmandu	Only larvae were recorded (in June)
Byanju *et al*. 2013 [93].	Bhakatapur, Nepal (April-September 2011)	Vector	Descriptive	Lymphatic filariasis vectors (*Culex quinquefasciatus*) reported above 2,000 m from Nagarkot with indoor higher than outdoor densities	No significant effects of month and climatic variables reported (but sampling only in warm months and method of data analysis is not explained in detail)
Dhimal *et al*. 2014 [94]	Nepal (September 2011-February 2012)	Vectors	Generalized linear models	Significant effects of climatic factors (temperature, relative humidity, precipitation) on the abundance of *Aedes aegypti* and *Culex quinquefasciatus* are reported	*Culex quinquefasciatus* is reported up to 2100 m asl and *Aedes aegypti* up to 1310 m in Kathmandu valley.
Dhimal *et al*.2014 [95]	Eastern Nepal (2012–2013)	Vectors	Longitudinal Entomological survey	The known malaria vectors in Nepal, *Anopheles fluviatilis*, *Anopheles annularis* and *Anopheles maculates* complex members were recorded from 70 to 1,820 m asl. Similarly, the vectors of chikungunya and dengue virus, *Aedes aegypti* and *Aedes albopictus*, the vector of lymphatic filariasis, *Culex quinquefasciatus*, and that of Japanese encephalitis, *Culex tritaeniorhynchus*, were found from 70 to 2,000 m asl in eastern Nepal. Furthermore, larvae of *Anopheles*, *Culex* and *Aedes* species were recorded up to 2,310 m asl.	The maximum altitude covered in the survey was 2,500 m asl and relationship with climatic factors was not determined in this study.
Joshi *et al*. 2006 [96]	Khotang district 2006	Visceral leishmaniasis	Case study	Autochthonous Visceral leishmaniasis case (10 yearl-old girl) from a Visceral leishmaniasis non-endemic district of eastern Nepal.	Referral case for diagnosis; the patient had no travel history to India or Visceral leishmaniasis endemic areas within Nepal.
Joshi *et al*. 2006 [54]	Nepal (1980–2003)	Visceral leishmaniasis	Descriptive	Increasing trend of Visceral leishmaniasis reported with majority of cases occurring during the rainy season and fewest during the winter.	Cased were confined to districts bordering the Indian state of Bihar
Pandey *et al*. 2011 [97]	Doti district 2011	Visceral leishmaniasis	Case study	The first autochthonous case of Visceral leishmaniasis(13-year-old male) from a VL non-endemic hilly district of western Nepal.	Referral case for diagnosis; the patient had no travel history to India or Visceral leishmaniasis endemic areas within Nepal.
Pun *et al*. 2011[98].	Nepal (April 1999-March 2009)	Visceral leishmaniasis	Descriptive	Increasing trend and geographic distribution of visceral leishmaniasis at a referral hospital from a non-endemic district	No classification of cases into autochthonous and imported ones
Scharz *et al*. 2011[99]	Achham district, Nepal (2011)	Visceral leishmaniasis	Case study	An autochthonous Visceral leishmaniasis case (17year-old woman) is reported from a Visceral leishmaniasis non-endemic hilly district of western Nepal.	Referral case for diagnosis; the patient had no travel history to India or Visceral leishmaniasis endemic areas within Nepal.
Pun *et al*. 2013[100]	Nepal (September 2010-October 2011)	Visceral leishmaniasis	Descriptive	Report of a series of locally transmitted autochthonous Visceral leishmaniasis cases from areas previously considered non-endemic, mostly in hill and mountain regions	Only referral cases in a tertiary care hospital in Kathmandu were included; the actual incidence of Visceral leishmaniasis in non-endemic areas can be expected to be many times higher
Uranw *et al*. 2013 [56]	Dharan, Sunsari district (2000–2008)	Visceral leishmaniasis	Outbreak investigation including case-control study	Report of urban transmission of Visceral leishmaniasis in Dharan city with a strongly clustered distribution	High chances of recall bias especially among control group; climatic variables not considered in analysis.

### Observed climate change in Nepal

Although analyses of observed temperature and precipitation data are still limited in Nepal, climate change effects are already occuring. Temperature data show a warming trend in Nepal. This warming trend is influenced by maximum temperatures with higher warming rates in the mountain regions compared to the lowlands of Nepal [61,66,71]. A recent study using the data of 13 mountain stations of Nepal (1980–2009) reported that only the maximum and mean temperatures are in an increasing trend without any changing trend of minimum temperatures [69]. In contrast, another study conducted in the Mustang district of Nepal’s mountain region using data between 1987–2009 shows an increasing trend of both minimum and maximum temperatures [72]. A general warming trend of all types of temperatures (minimum, maximum and mean) is observed in Nepal with a higher warming rate in mountain and hill regions compared to lowland (Terai) regions when the data of eight stations between 1971–2006 are analysed [64]. The annual cycle of temperature lapse rate shows a bi-modal pattern with two maxima in the pre- and post-monsoon seasons as well as two minima in winter and summer. This is completely different from other mountain regions suggesting different contributing factors in individual seasons[67]. Hence, the trend of temperatures, especially of minimum temperatures, depends on the location of meteorological stations because of large micro-climatic [67]. Precipitation does not show a distinct trend in Nepal. The analysis of precipitation data from 78 stations all across Nepal (1948–1994) and sub-regional records (1959–1994) reveal great interannual and decadal variability in precipitation but do not show a long-term trend [62]. The analysis of data from 26 stations (1961–2006) shows an increasing trend of extreme precipitation events in total and heavy precipitation at most stations [64]. A strong relationship between rainfall and elevation in the pre-monsoon and post-monsoon is observed along the Himalayas of Nepal [68]. Interestingly, two significant rainfall peaks are found over the southern slope of the Himalayas (between 500–700 and 2,000–2,200 m above sea level [asl]) during the monsoon season whereas relatively large amounts of rainfall occur over higher elevations during the pre-monsoon season [68]. Spatial and temporal variation in precipitation pattern and significant roles of mountainous relief in yielding localized precipitation patterns is reported in Nepal [63].

However, changes in the extreme events, consistent with climate change effects, are more significnat in Nepal. A declining trend of cool days and inclining trend of warm days are observed in the higher altitudes of Nepal [64]. The combined effects of increased temperature and diminished snowfall followed by rapid shrinking of the majority of glaciers have already resulted in a reduction of water available for drinking and farming in the mountain regions of Nepal [66,72,101]. The precipitation extremes show an increasing trend in total and heavy precipitation albeit no systematic difference is observed in extreme precipitation trends beween the highlands and lowlands [64].

The Hadley Centre's high-resolution regional climate model PRECIS (Providing Regional Climates for Impact Studies) projects significant warming towards the end of the 21^st^ century and a decrease in monsoon precipitation over the Central Himalayan region (which includes Nepal) during the period 2011–2040 and an increase in seasonal rainfall during the period 2071–2098 compared to the baseline period (1961–1990) [70]. In contrast, other studies with regional climate models such as COSMO-CLM project over 70% decrease in monsoon rainfall in parts of northern India at the end of this century [65] and because of higher evapotranspiration rates with higher temperature, a decreasing amount in water availability has also been reported. The fifth assessment report of IPCC conclude that different climate models have varying degree of success in simulating past mean state andclimate variability compared to observed sations data and large uncertainities in climate change projecttions are reported as estimated in multi-model ensembles (especailly for precipitation) [1]

### Spatiotemporal distributions of vector-borne diseases in Nepal

#### Malaria

**Fig 3:**
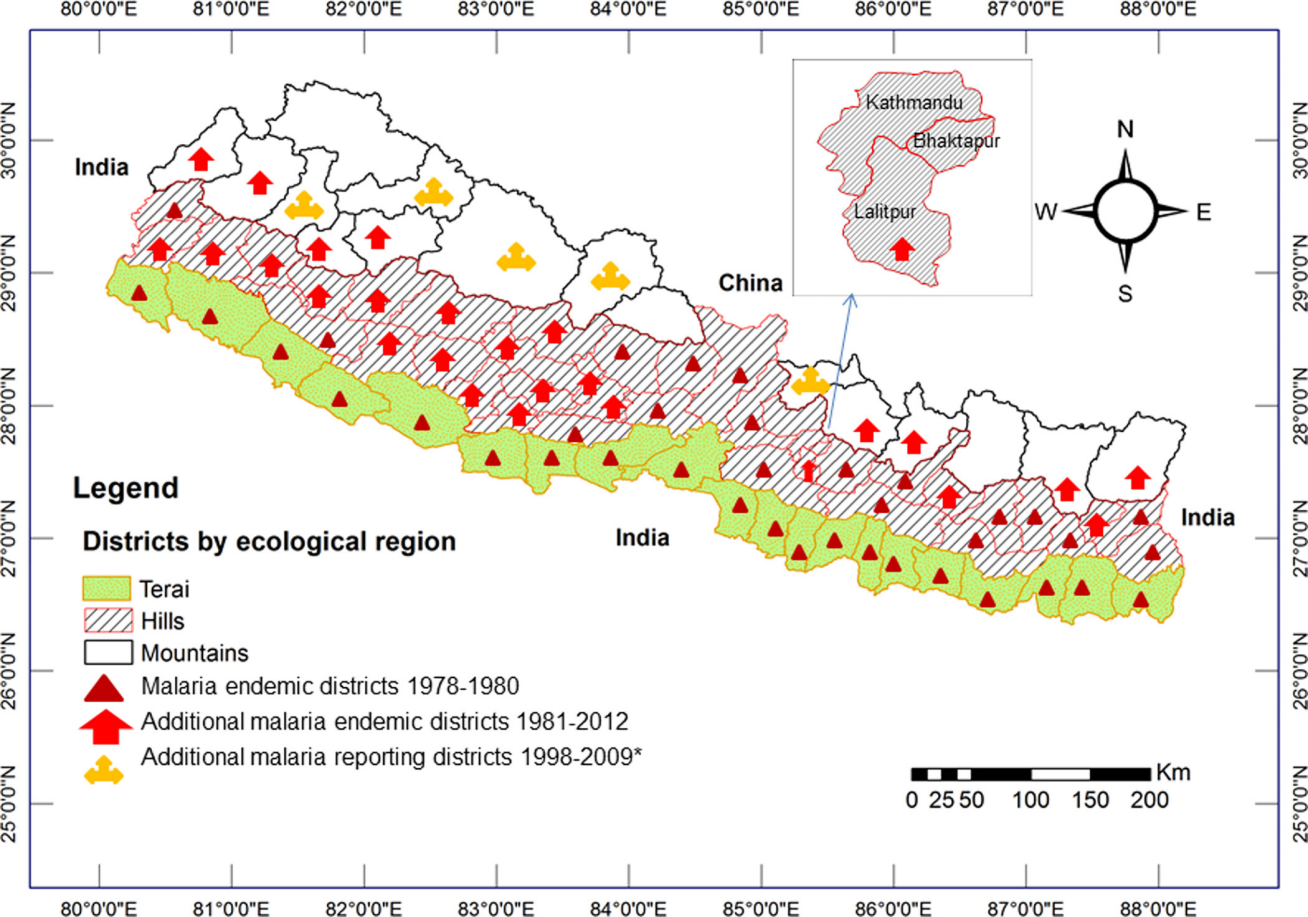
Spatiotemporal distribution of malaria in Nepal (1978–2012). The active case detection of malaria in Nepal between 1978 and 1980 recorded autochthonous malaria cases from 38 districts of the Terai and hill regions (< 1,200 m above sea level). Autochthonous malaria cases were recorded from 26 additional districts of Nepal between 1981 and 2012; these numbers also include malaria cases from mountain regions. The symbol (*) indicates that the classification of reported malaria cases, i.e., as autochthonous or imported, is not known.

In Nepal, a heterogeneous spatial distribution and fluctuating trend of malaria incidence has been reported with a higher incidence in southern districts bordering India [90]. The distribution of the disease, which was previously confined to the forest and forest fringe regions of the Terai lowlands and so-called Inner Terai valleys and hills (<1,200 m asl) in 38 districts [86], is now observed to extend to the hills and mountains (> 2,000 m) [87] and malaria is now endemic in 65 out of the 75 administrative districts [89,102]. Fourty four species of *Anopheles* mosquitoes have been recorded in Nepal and out of these, seven have been incriminated in malaria transmission (*Anopheles minimus*, *An*. *fluviatilis*, *An*. *maculatus*, *An*. *dravidicus*, *An*. *pseudowillmori*, *An*. *willmori* and *An*. *annularis*). They were observed at least 2,000 m asl [95] and cause seasonal malaria epidemics in Nepal, including in areas above 2,000 m [51,52]. During the last decade, the incidence of confirmed malaria cases has declined significantly in Nepal following the introduction of the free distribution of long-lasting insecticide impregnated bed-nets (LLINs) and artemisinin combination therapy (ACT) for the treatment of *Plasmodium falciparum* malaria [89]. However, the proportions of *P*. *falciparum* and imported malaria cases have increased considerably in comparison to the total number of confirmed malaria cases [89]. This implies the possibility of a gradual shift in the *Plasmodium* parasite population possibly due to the rising temperature trends. Moreover, indigenous cases of *P*. *vivax* malaria and *P*. *falciparum* infections have been reported from the hill and mountain regions of Nepal in later years. A positive relationship between rainfall and malaria cases with a certain time lag has been observed in Nepal [87]. A significantly positive correlation of malaria incidence with minimum as well as maximum temperatures and rainfall was found in a study conducted in the high-endemic malaria district of Jhapa [88]. However, non-climatic variables were not included in the analysis in that study, and the climatic variables assessed were not significant predictors of malaria incidence in time series analysis. Another recent study shows that a1°C increase in minimum and mean temperatures increased malaria incidence by 27% and 25%, respectively. The reduction in malaria incidence was 25% per one unit increase of LLINs [23]. The spatiotemporal distribution of malaria in Nepal (1978–2012) is presented in Fig 3 [50,86,90,103].

#### Dengue

**Fig 4:**
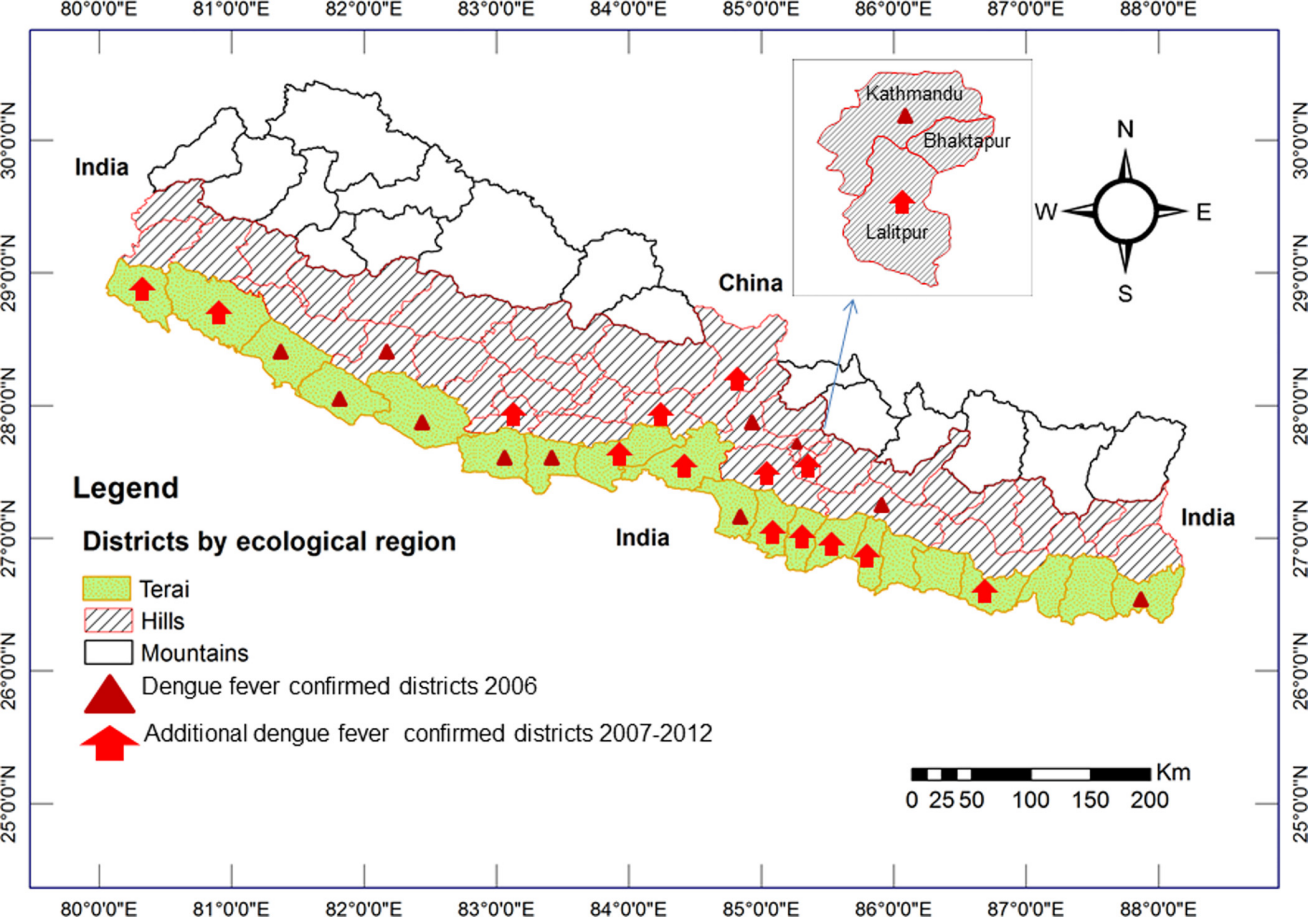
Spatiotemporal distribution of dengue fever cases in Nepal 2006–2012. Autochthonous dengue fever cases were recorded from ten districts of Nepal during the first outbreak in 2006. The travel history of dengue fever cases reported from Kathmandu in 2006 was not known. However local transmission of dengue virus and the presence of the primary dengue virus vector *Aedes aegypti* were confirmed from additional 10 districts of Nepal including Kathmandu between 2007 and 2012.

The first reported case of dengue virus (DENV) infection in Nepal was a Japanese volunteer in 2004 [73], and the first local transmission of DENV in Nepal was confirmed at the beginning of an outbreak in 2006 (August-November) in lowland urban areas of 11 districts [58,104]. During this outbreak, the first record of the primary vectors of DENV, *Aedesaegypti* mosquitoes, and the presence of all four serotypes of the virus (DENV1-4) were reported [58]. Previously, no *Ae*. *aegypti* had been reported in Nepal, but the secondary vector of DENV, *Ae*. *albopictus* was known to have existed in the lowlands and hill regions including Kathmandu (the capital city of the country located above 1,300 m asl) as early as the 1950s [52,91]. Since the first DF outbreak in 2006, DENV and its vector *Ae*.*aegypti* have been rapidly expanding across the country including the densely populated Kathmandu valley and mountain regions of Nepal[57,59,74,75,94,95]. During an epidemic in 2010, 917 DF cases including five deaths were reported[50]. During this outbreak, DF cases started to be recorded at the beginning of August (monsoon season) in a hilly district (~360 m asl), rapidly spread in lowland Terai districts (90 m), appeared in the hill districts of Dhading (> 900 m) and Kathmandu (> 1,300 m) in October with a peak in November (post-monsoon season) and diminished in mid-December (winter season) [59]. The study of the 2010 epidemic also recorded *Ae*. *aegypti* in all affected areas and provided data suggesting that DENV isolated from Nepalese patients was phylogenetically close to Indian DENV pointing to an import of the virus from India. Significant effects of the climatic factors temperature, rainfall and relative humidity, physiographic region and month of collection on the abundance of adult *Ae*. a*egypti* were reported in a recent study from central Nepal [105]. The spatiotemporal distribution of DF cases in Nepal in the period 2006–2012 is shown in Fig 4 [58,59,75,104].

#### Visceral leishmaniasis (VL)

**Fig 5:**
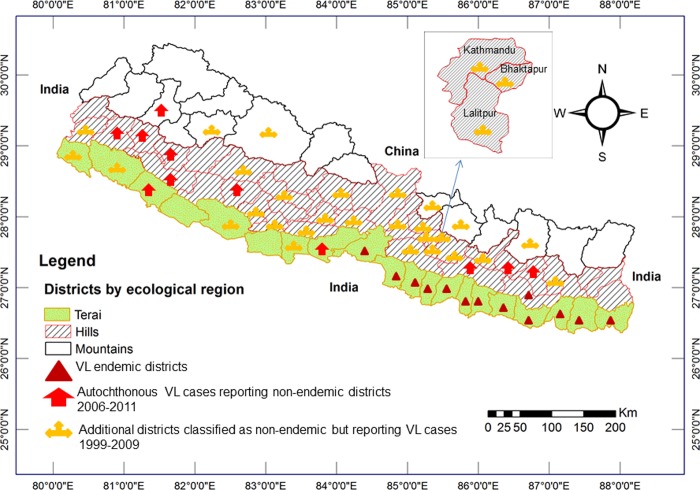
Spatiotemporal distribution of visceral leishmaniasis cases in Nepal (1980–2011). Before 2006, visceral leishmaniasis (VL) was endemic only in 13 lowland districts of the Terai region bordering Bihar state, India. Between 2006 and 2011, autochthonous VL cases were reported from 11 additional districts mostly in the hills but including one in the mountains. Moreover, VL cases were reported from 25 additional districts but their origin (i.e., autochthonous or imported) is not known.

In Nepal, VL cases were first recorded in 1980. At that time confirmed cases were confined to lowland Terai districts of eastern and central Nepal that border India’s state of Bihar, followed by records from 13 endemic districts and an increasing trend of incidence until 2003 [54]. Despite a declining trend of VL incidence in Nepal after 2003, VL is now increasingly reported from districts classified as non-endemic amounting by 2009 to 47 out of the 75 districts of Nepal (albeit cases were not classified as indigenous or imported) [98]. Although the disease was previously assumed to be confined to rural households with damp earthen floors and especially to poor families, autochthonous VL cases have since 1997 also been reported among residents of the urban area of Dharan city with highly clustered distributions [56]. Disease transmission in Dharan was confirmed by PCR identification of both vector, the sand fly *Phlebotomus argentipes*, and parasite, *Leishmania donovani*, inside town [56]. Moreover, series of autochthonous VL cases are now being reported from new areas mostly in hill and mountain regions of Nepal which had previously been considered to be non-endemic for this disease [96,97,99,100]. A positive association of VL cases with temperature and rainfall has been observed with reports of disease outbreaks 2–3 months after heavy rainfall in Nepal [87]. The abundance of the vector *P*. *argentipes* has also been found to be positively correlated with the maximum temperature of the month of collection and negatively correlated with the precipitation of previous months in both Nepal and India [106]. The spatiotemporal distribution of VL cases in Nepal (1980–2011) is shown in Fig 5 [54,96,97,98,99,100].

#### Japanese encephalitis (JE)

**Fig 6:**
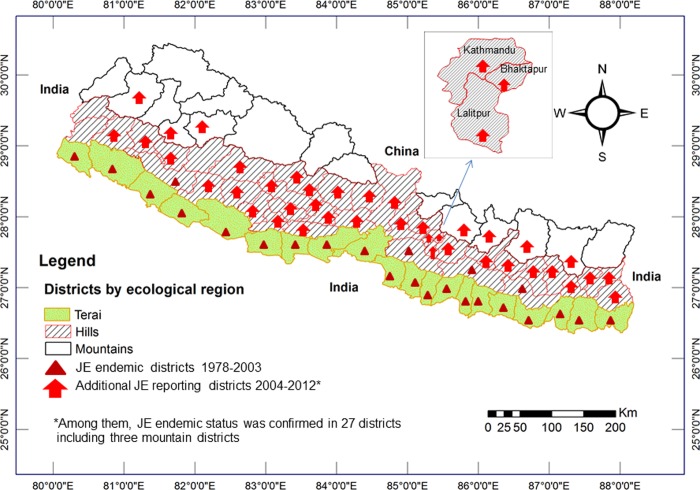
Spatiotemporal distribution of Japanese encephalitis cases in Nepal (1978–2012). Japanese encephalitis (JE) cases were recorded only from 24 districts of the lowland Terai between 1978 and 2003 in Nepal. After the start of surveillance for acute encephalitis syndrome with the support of the World Health Organization (WHO) in May 2004, JE cases were reported from 40 additional districts including mountain regions between 2004 and 2012. Among these 40 additional districts, JE endemicity was confirmed for 27 districts including three mountain districts.

Infections with Japanese encephalitis virus (JEV) moved northward in India and began to be seen in Nepal in the late 1970s [76] when epidemics occurred in lowland districts bordering India in western (Rupendehi) and eastern Nepal (Morang) in 1978 [78]. The mosquito species *Culex tritaeniorhynchus*has been reported to be the principal vector of JEV in many parts of Asia including Nepal [52,81]. It was first recorded in Nepal in 1965 with a distribution ranging from the lowland to hill regions including Kathmandu valley [60]. Although most reported JE cases in Nepal were initially confined to 24 districts in the lowland Terai [78], JEV transmission is now established in hill and mountain districts of Nepal, including Kathmandu valley, which were previously considered non-endemic for this disease [77,79,80,81,82]. Moreover, there are reports of spatial cluster of JE incidence with a shift from the Terai lowlands to hill and mountain regions after 2005 [81]. The risk of JE was also associated with paddy field configuration at the landscape level [83]. A presence of mosquitoes in pig farms and their association with JE sero-positivity has been reported from four mountain districts of Nepal [82]. A significantly positive association of JE incidence with monthly temperature and the percentage of irrigated land, and a negative association with low precipitation has also been reported from Nepal [81]. The spatiotemporal distribution of JE cases in Nepal (1978–2012) is shown in Fig 6[77,79,80,81,82,107].

#### Lymphatic filariasis (LF)

**Fig 7:**
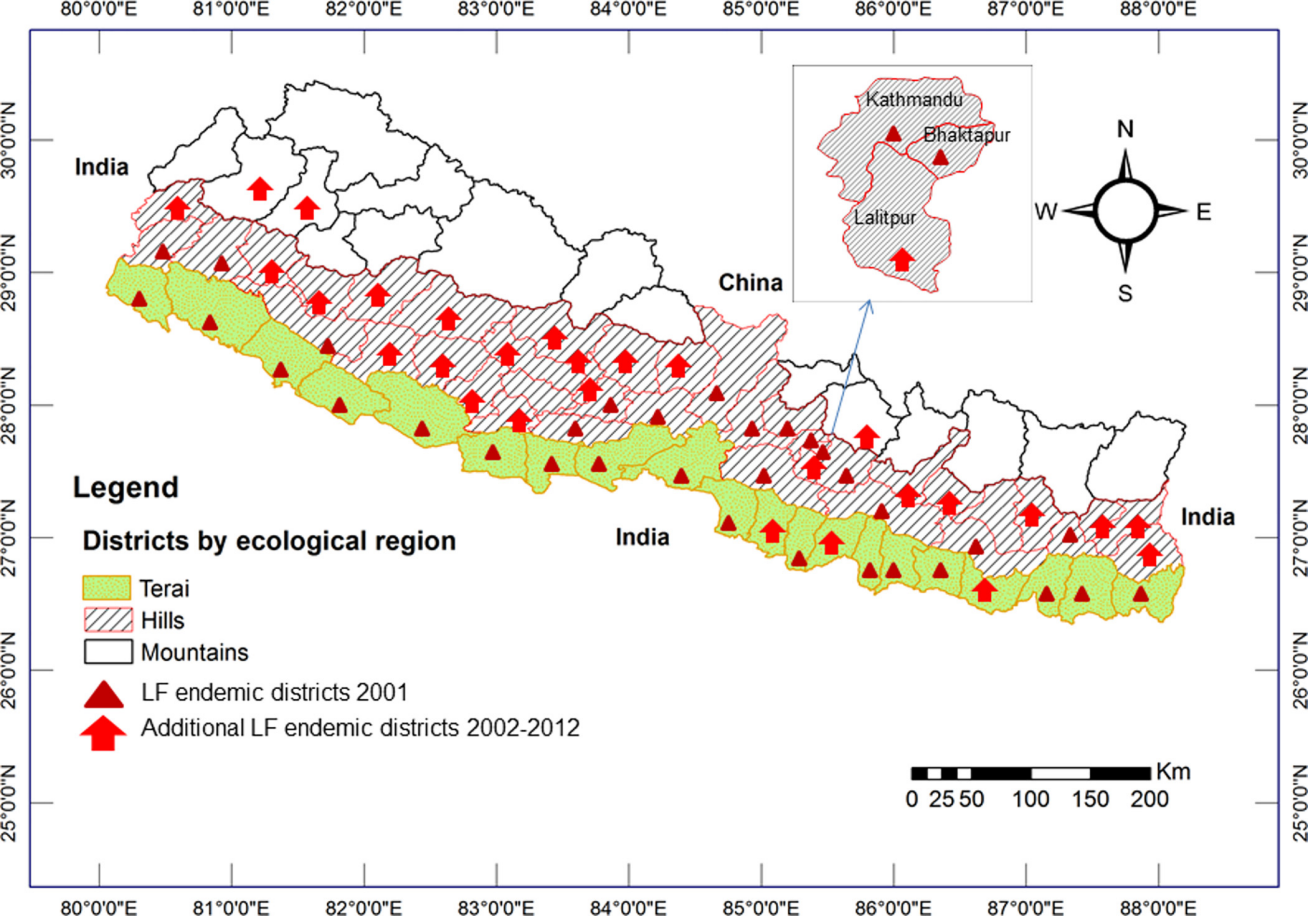
Spatiotemporal distribution of lymphatic filariasis in Nepal (2001–2012). In 2001, lymphatic filariasis mapping using immunochromatographic card tests in 37 districts of Nepal showed that LF was endemic in only 33 districts. Between 2002 and 2012, LF was confirmed as endemic in 60 districts of Nepal including mountain region districts.

The mosquito species *Culex*. *quinquefasciatus*, the principal vector of *Wuchereria bancrofti* microfilaria in South Asia, was first recorded in Nepal in 1956 [91] and found to occur within the LF endemic zones of this country[50,52,53]. In the year 2001, LF was endemic in 33 out of 37 surveyed districts of Nepal. The majority of cases were confined to an altitudinal range between 500–700 m asl, however, with a substantial number of cases at altitudes between 900–1400 m asl[84]. A sentinel surveillance conducted in 2007 among 7,000 people residing in six districts of the lowland (Terai), hill and mountain regions of Nepal reported the highest microfilaria infection rate (2.0%) in the mountain district of Sidhupalanchowk [85], suggesting a shift of LF transmission to the mountain region of Nepal after the introduction of mass drug administration (MDA) programmes in lowland and hill districts which had started in Parsa district in 2003. By 2013, six rounds of MDA had been completed in 16 endemic districts and four, three, two and one round of MDA in 10 districts each, and a gradual expansion of MDA reached 5 endemic districts covering 74% of the total population at risk (N = 21,852,201) [50]. However, 61 out of 75 administrative districts have already been reported as being LF endemic, and Nepal plans to cover the remaining six endemic districts with MDA by 2014 and achieve <1% prevalence in all endemic districts by 2018 [50]. Previously, *Cx*. *quinquefasciatus* mosquitoes had been recorded in all endemic districts ranging from 90 to 1,800 m asl[102], and recent studies report the distribution of *Cx*. *quinquefasciatus*up to at least 2,100 m (the highest sampled altitude in that study) in the districts of Dhunche and Rasuwa which had previously been regarded as non-endemic for LF [94], and above 2,000 m in Nagarkot of Bhaktapur district [93]. Moreover, significant effects of the climatic factors temperature and relative humidity, physiographic region and month of collection on the mean abundance of *Cx*. *quinquefasciatus*per (per trap) were found [94]. The spatiotemporal distribution of LF in Nepal (2001–2012) is shown in Fig 7[84,85,102]

## Discussion

The review of observed and future projections of climatic data show a conducive environment for the transmission of VBDs in Nepal, especially in the highlands (mountains) which had been assumed to be free from these diseases. Despite a decade-long armed conflict and political instability in Nepal, there has been a substantial decline in the incidence of all major VBDs except DF which has only emerged in Nepal since 2004. The presence of disease vectors and reports of series of autochthonous cases of VBDs in hill and mountain regions of Nepal that had previously been considered to be non-endemic suggests that the local transmission of VBDs might be favoured by rising temperatures. However, the transmission of VBDs among humans is more complex than mere temperature changes, and this fact has been extensively reviewed [9,28,33,34,39,108,109,110,111]. One may also hypothesize that improvements in diesease surveillance and health care services, land use changes, population growth, globalization in general, in particular international trade, tourism and travel, migration and other movement of people, the expansion of road networks and the shipment of goods, unplanned urbanization and improvements in livelihood and access to health care services, etc., could be responsible for an increased detection of VBD cases in new areas. However, the presence of vectors and local transmission of VBDs at altitudes above 2,000 m, which clearly stands against the conventional logic that high altitude regions are free of VBDs because of cold temperature, strongly suggests that global warming is playing a role in the observed transmission. Therefore, we discuss below climate change and the spatio-temporal distribution of VBDs in Nepal in comparison with the results of studies from other mountainous countries around the world.

A warming trend of annual mean temperatures is observed throughout the country indicating that climate change is already occuringin Nepal. However, large spatial and temporal variation in the trends of minimum and maximum temperatures is observed across different meteorological stations. The warming signal is clearer for maximum temperature with a more pronounced warming in the mountains compared to the lowlands ofNepal. This is in sharp contrast to the warming trend of the Tibetan Plateau where the minimum temperature is increasing at a faster rate than the maximum temeprature [112,113,114]. but consistent with the trend observed in the western Himalayas of India [115,116], indicating a role of the Indian monsoon in the regulation of temperature through complicated feedback. Increasing trends of both minimum and maximum temperatures with greater warming rates in higher elevations have also been reported from the Rocky Mountains in Colorado, USA [117].

Precipitation is one of the major climatic factors affecting transmission of VBDs. The absence of a distinct long-term trend in precipitation changes in Nepal despite increasing GHG and strongly increasing aerosol concentrations in the region (especially through the neighbouring countries India and China) might be explained by a moister but less intense monsoon circulation [65]. Nepal’s precipitation is affected by two major air movements: the summer monsoon which originates from the Bay of Bengal in the east, and the winter western disturbances which affect mostly the western parts of the country and result in snowfall in the mountains. A large spatial variation in annual rainfall over Nepal, ranging from less than 150 mm to more than 5,000 mm, is observed and is largely associated with the South Asian monsoon [118]. About 80% of the annual precipitation occurs during the monsoon season (June-September) followed by the post-monsoon season (12.7%) [118]. A positive correlation has been shown between the all-Nepal precipitation and the Southern Oscillation Index (SOI) series suggesting a strong association between the El Niño Southern Oscillation (ENSO) and precipitation fluctuation in Nepal [62]. The all-Nepal precipitation records do not agree well with the all-India precipitation record but resembles that of the northern part of India. This means that the precipitation climatology of the Himalayan region and adjacent areas differs greatly from the southern part of the Indian subcontinent. As a result, aggregated precipitation data from all over India cannot provide a valid representation of the entire subcontinent [62]. Although the regional climate models show an increasing trend of temperatures and no distinct trend in precipitation amount, most regional climate models report too warm temperatures in the northern parts of India that are too high compared to the observations, and the amounts of precipitation and its spatial distribution differ significantly between the regional climate models [119].

The observed declining trend of cool days and increasing trend of warm days in the higer altitudes of Nepal [64] is consistent with the global trend [43,44]. However, a multi-country study carried out in South Asia showed only the extreme temperature indices of low altitudes and latitudes to be consistent with general warming whereas stations at higher altitudes and latitudes showed both positive and negative trends, suggesting that high-elevation sites might be more influenced by local environmental factors making projections of future developments difficult [120]. On the other hand, a study from Nepal suggests that the contribution of the effects of urbanization and local land use changes to the all-Nepal temperature change is minimal, indicating that global warming is influencing the warming trend in Nepal which is comparable in magnitude with regional and northern hemisphere temperature trends [61]. It has also been argued that the diminishing snow and glacier covers in the mountain regions of Nepal will change the surface albedo of the region, which in turn will increase surface temperature, thereby acting as a positive feedback mechanism resulting in higher warming rates of maximum temperatures in the mountains compared to the lowlands [61,121]. The decreasing trend of the observed maximum temperature in the winter season of the lowland Terai regions is believed to be due to the occurrence of cold waves and prolonged periods of fog which have become more prominent in the last decades [64,118].

A review of elevation-dependent warming and its possible causes in four high mountain regions–the Swiss Alps, the Colorado Rocky Mountains, the Tibetan Plateau/Himalayas, and the Tropical Andes–showed variation in the trends of extreme temperatures suggesting the need for a comprehensive study analysing the minimum and maximum temperatures separately for all mountain regions together to better understand elevation-based warming in mountains [122]. The highly varied topography over short distances and poor coverage of observational datasets especially in mountain regions render spatiotemporal climate change projections for Nepal difficult and any projections must be interpreted with caution [123]. Nevertheless, uncertainty in climate projections should not be a barrier for assessing vulnerability, impacts and adaptation options.

Several studies predict an increasing trend of the epidemic potential and the transmission season of malaria in temperate regions due to climate change [14,18,19,20,21]. The duration of the malaria transmission window in India is predicted to increase in the northern and western states, and shorten in southern states under different climate change scenarios[124]. Accordingly, the temperate region of Nepal is predicted to be at risk of malaria due to climate change because it is experiencing a much higher warming trend compared to the sub-tropical regions, and sporadic autochthonous cases of malaria have been reported from mountain regions previously considered free of malaria risk. Reports of autochthonous cases of malaria in the highlands may be due to an establishment of vectors above 2,000 m asl (i.e., the altitudinal range might have increased) [51,52], continuous import of malaria cases due to an increasing movement and migration of people [50,89]and may be favoured by rising temperatures because *P*. *falciparum* has a higher temperature requirement than *P*.*vivax* (sporogonic temperature threshold for *P*.*vivax* is generally assumed to be 14.5–16.5°C and for *P*. *falciparum*16.5–18°C [21]. As Nepal is preparing to move toward malaria elimination with the ambitious goal of achieving this by 2026, the surveillance of malaria in areas previously considered to be low or no-risk areas should be strengthened. The malaria control in mountain regions is much more difficult compared to the lowlands owing to geographical difficulty, a scattered human population, poor coverage of health services and the fact that the majority of malaria cases in these regions are caused by *P*. *vixax* which has a high relapse/re-infection rate in Nepal [125].

Although the first autochthonous case of DENV in Nepal was confirmed at the beginning ofthe first outbreak in 2006, the case of a DENV infection in a Japanese volunteer to Nepal in 2004 [73] suggests that DENV was already being transmitted in Nepal prior to 2006. The rapid expansion of both DENV and its primary vector *Ae*. *aegypti* across the country, including mountain regions, within a short period of time[57,59,73,94,104,126,127] may be attributed to an introduction of mosquito eggs in used tyres, transport of adult mosquitoes in motor vehicles, and increased domestic and peri-domestic breeding opportunities due to increasingly frequent and long-lasting water shortages creating a water storage culture in urban areas like the Kathmandu valley. It may be further enhanced by rising temperatures and an influx of DENV via infected people who travelled to dengue endemic countries, e.g., India and other Southeast Asian countries [59]. The rapid expansion of dengue vectors in Nepal after 2006 may have been further supported by the end of the decade-long armed conflict in Nepal (1996–2006). After the end of the armed conflict in 2006, rapid urbanization, road expansion, trade and business and mobility of people increased in Nepal, all of which might have driven the rapid expansion of DENV in Nepal. The suppression of DF outbreaks between 2006 and 2010 may have been associated with a rapid consumption of old tyres (the major breeding containers of dengue vectors) in the major urban areas mostly of the Terai lowlands which were routinely burnt during frequent strikes and protests. A geographical expansion of DENV in this decade, since 2004, also occurred in another Himalayan country, Bhutan [128]. The expansion of DENV vectors towards mountain region in Nepal is consistent with findings from the Eastern Himalayas [129] and highlands of Mexico [130]. First autochthonous cases of chikungunya virus (CHKV) infection (also transmitted by *Ae*. *aegypti* and *Ae*. *albopictus*) were reported in Nepal in 2013 [131] and a CHKV outbreak in Bhutan in 2012 [132]. Consistent with the expansion of DENV and CHKV in temperate regions of South-East Asia, expansions of the ranges of the disease vectors and an increasing number of autochthonous cases of DENV and CHKV infections have been reported in Europe where both climate change and non-climatic factors have been reported as contributing factors [133,134]. The low knowledge of people on DF prevention and control in Nepal [105,135] coupled with weak diagnostic facilities, a poor case reporting system which does not incorporate the private healthcare sector, a lack of routine vector and national DF surveillance, and poor multi-sector coordination for DF control [75,136] can intensify DENV expansion and epidemics in Nepal.

Resistance development to first-line drugs against VL and the inadequate implementation of vector control interventions have been reported as the major causes for this increasing trend of VL in Nepal [54,137,138]. Important risk factors that promote VL transmission are poverty, housing styles (i.e., mud walls and damp floors in houses), the presence of cattle, and peri-domestic vegetation [139,140,141,142,143]. As only patients with disease symptoms are eligible for treatment and many asymptomatic cases hidden in the community, the treatment of cases had almost no effect, in contrast to vector control, suggesting the need of effective vector control interventions [144]. Although a declining trend of VL in Nepal has been observed after 2003, the disease is mostly reported from endemic districts only by public health institutions while an increasing trend of VL cases from non-endemic districts has caused worries about its control and elimination in Nepal. The positive correlation between the abundance of the VL vector *P*. *argentipes* and the maximum temperature of the month of collection in Nepal and India [106] as well as the positive association between VL cases and annual rainfall and temperature [87], indicate an effect of climatic factors on VL transmission in Nepal. Impacts of climatic variability on the occurrence and transmission of leishmaniasis have been reported in many studies [27,145,146,147]. Hence, the recently observed expansion of VL into new areas of the country may be facilitated by climate change along with other factors and constitutes an obstacle to achieving the VL elimination goal by 2015.

As the reported major environmental factors influencing JEV transmission in Asia including Nepal are temperature and precipitation [81,148,149,150,151], climate change along with the JE vaccination campaign which started in 2006 the southern districts in Terai[79,81,152,153,154] may have already affected the spatial and temporal distribution of JEV transmission in this country. The presence of the principal vector of JEV, *Cx*. *tritaeniorhynchus*, and JEV circulation in higher altitudes of Tibet have also been reported [155]; this is consistent with reports of JEV transmission in the mountain regions of Nepal [79,80]. However, clinical cases presenting with non-malarial febrile or encephalitic syndromes are reported in increasing numbers every year in Nepal but their etiology is not known in the absence of studies on viruses other than DENV and JEV. For example, the isolation of Kunjin viruses (Australasian subtypes of West Nile Virus [WNV]) from the sera of domestic animals from districts with a low prevalence of JEV has suggested that WNV, too, may circulate in Nepal [156] and recent study shows evidence of the continued spread of WNV in Nepal[157]. Recently, outbreaks of Nipah virus have been suspected in eastern Nepal but still await laboratory confirmation.

The establishment of *Cx*. *quinquefasciatus* mosquitoes already above 2,100 m asl[94,127], frequent movement of people between endemic and non-endemic areas, low acceptance of MDA due to severe adverse effects in some people, and its low coverage in urban areas [102] pose challenges for achieving LF elimination in Nepal by 2020. In the future, this disease is likely to expand in mostdistricts of Nepal if integrated vector-control measures and active disease surveillance are not implemented. Although studies for the Indian sub-continent were not found, model projections show that climate change and population growth are dominant factors for predicting the risk of infection and spread of LF on the African continent [30,158]. However, predicting the transmission risk of LF under different climate change scenarios is difficult owning to the chronic nature of this disease and its association with the standard of living of people and environmental sanitation.

Although socio-economic development, medical care and vector-control measures can outweigh the influence of climate change on VBDs in some areas [13,159,160]., the worst effects of climate change will occur in the poorest and most vulnerable regions least benefitted by economic growth [46,159,160]. Accordingly, with the improvement in economic status and overall health indicators in Nepal, the reported incidence of all VBDs in Nepal has declined but reports of confirmed autochthonous cases of VBDs from new areas including mountain regions that had previously been considered non-endemic is worrisome. Against a background of weak health care systems, difficult geographic terrain, lack of vector-control interventions in the highlands, and continuous influx of infected people from disease endemic areas, climate change can intensify the potential risk of VBD epidemics in the mountain regions of Nepal. However, our review is not conclusive for a causal relationship between climate change and VBDs and need further research to determine attribution to climate change. Hence, it calls for further research using long-term data records and controlling possible confounders in analyses. The findings of our review are consistent with similar reviews on climate change and VBDs from neighboring countries like India and China as well as others [9,10,161,162]. These studies also suggest that the available data are inadequate for conclusions on the impacts of climate change on VBDs because of complex relationships with non-climatic factors and inconsistent findings in different geographical regions. Thus, we propose the following specific future research priorities taking into account the combined effects of climatic and non-climatic variables on disease vectors and VBDs:

- Entomological, virological and parasitological research in different transects along an altitudinal gradient across the country to determine the presence of vectors and their role in disease transmission
- Development of VBD risk maps for Nepal based on entomological, virological and parasitological evidence and climatic as well as land use to better guide the allocation of limited resources to the most vulnerable groups
- Social-ecological and socio-economic research to identify the adaptation needs in different ecological regions and settings and plan public health preparedness, taking into account ethnic, religious, cultural and gender differences

## Conclusion

The studies reviewed here suggest that both the observed and projected climate are conducive for the transmission of VBDs in the mountain regions of Nepal which had previously been considered non-endemic for these diseases. The short-term data shows a clear association between climatic factors and VBDs, but it is complex and difficult to project long-term effects of climate change in the face of rapid environmental and socio-economic changes and attribution to climate change is not determined in the existing studies. Despite continuous efforts of the government to control them and their declining incidence over the last decade (except for DF), VBDs have over the years been expanding their geographical ranges especially in mountain regions of the country. This might be attributed to environmental changes, in particular climate change, along with socio-economic factors. However, the observed spatial expansion of VBDs in new areas, especially in cool margins of mountain regions, that is correlated with the observed warming climate does not necessarily show a causal relationship. As VBDs show a heterogeneous distribution and spatiotemporal variation in the trends of climatic variables across the country, well-designed long-term local studies are needed to determine attribution of climate change to the observed transmission and distribution of VBDs in new areas. Therefore, VBD monitoring, surveillance and research should be strengthened in areas where risk of VBD is not yet determinedand VBD control programmes are not yet focused. Moreover, tourists and returning migrant workers coming to Nepal from disease endemic regions (including the country’s own lowlands) should be made aware about VBDs, their responsibility and potential role in spreading infections especially when travelling in mountain regions, and should be encouraged to engage in reasonable preventive and prophylactic measures including vaccination.
